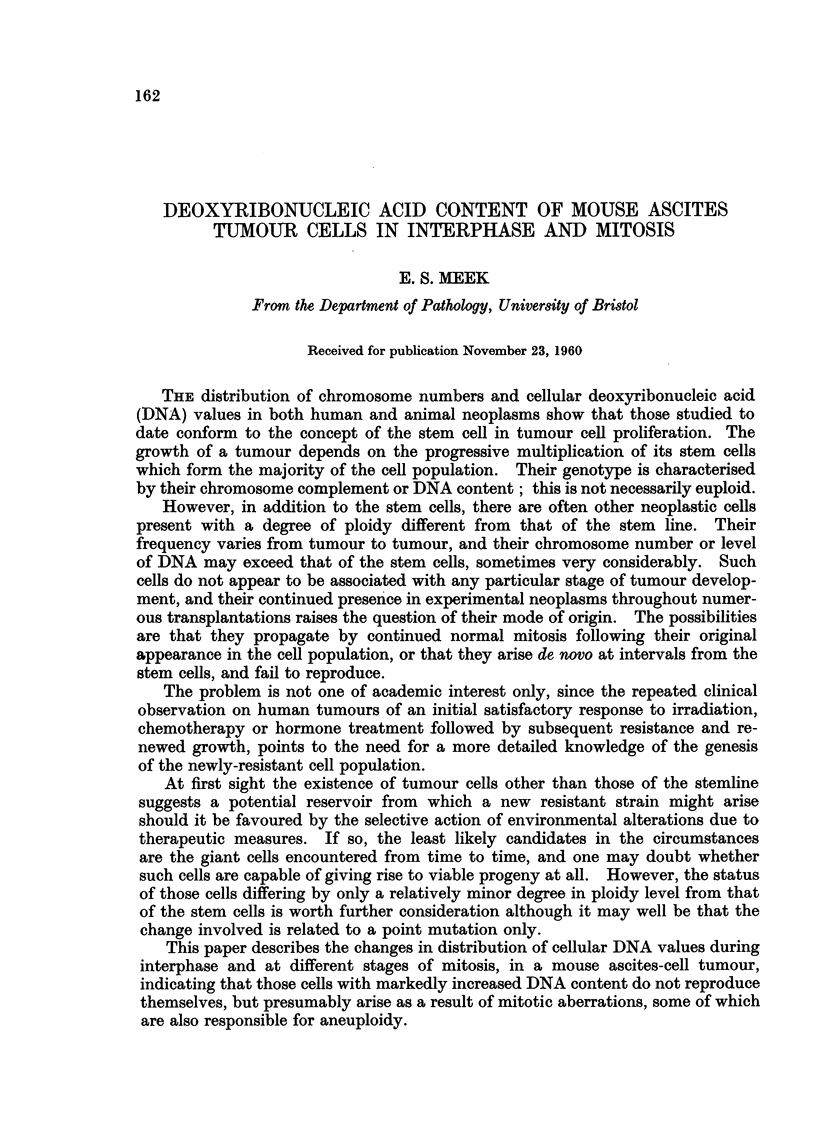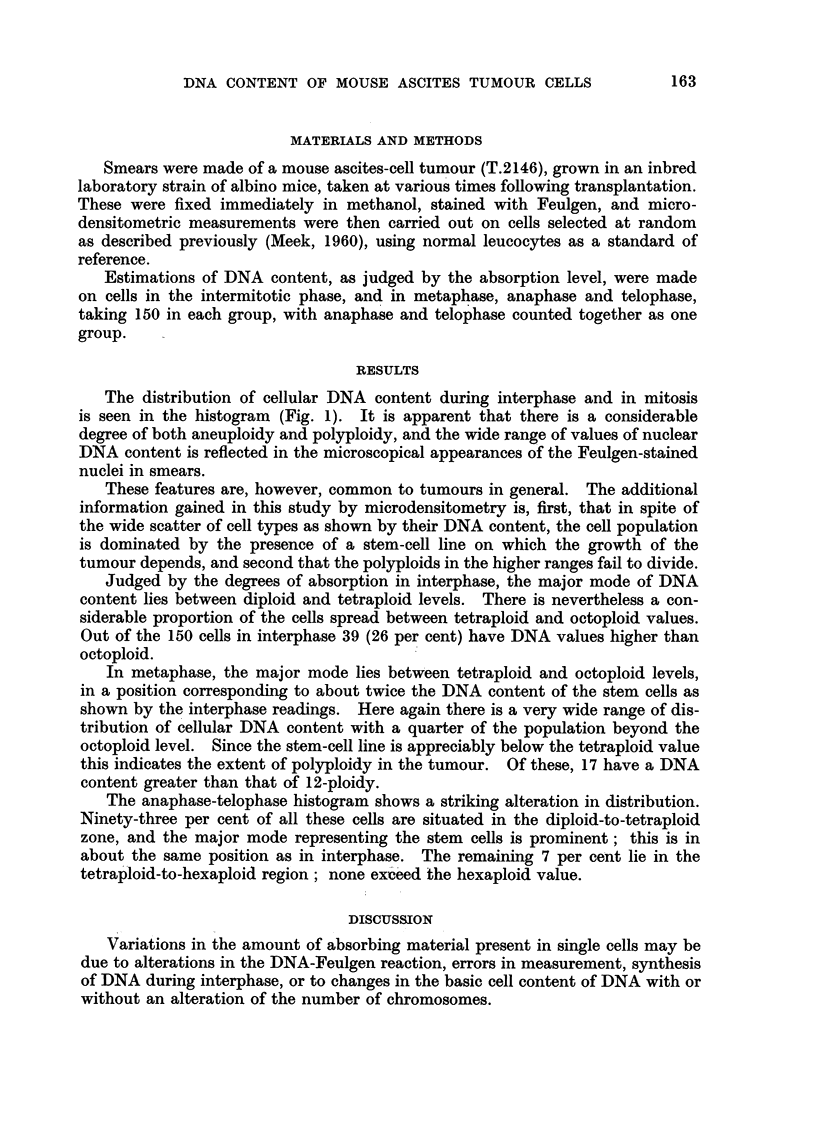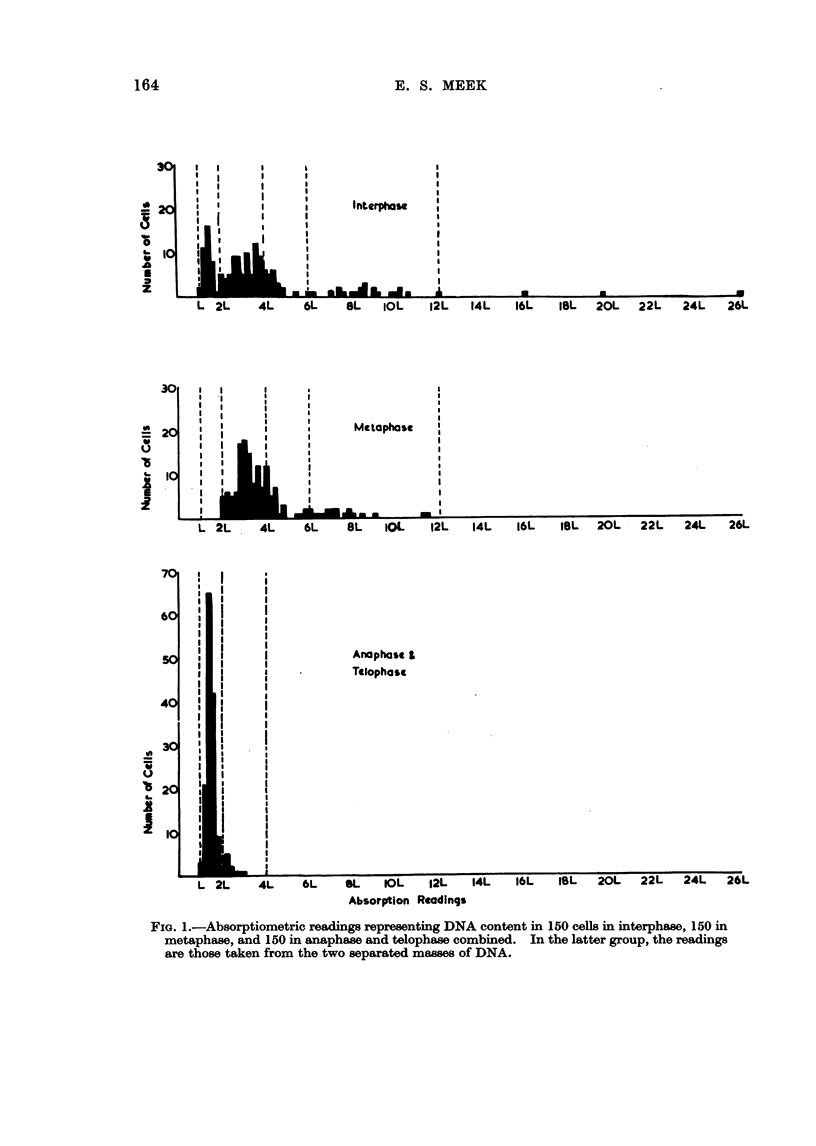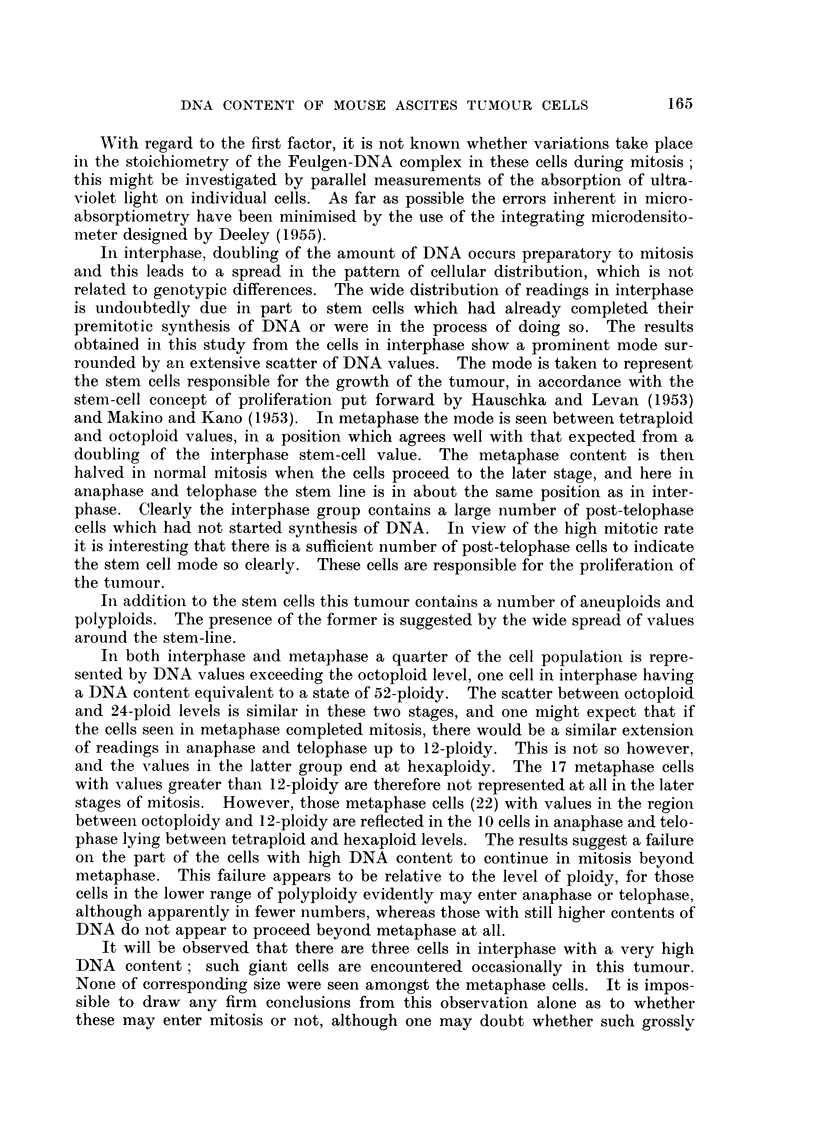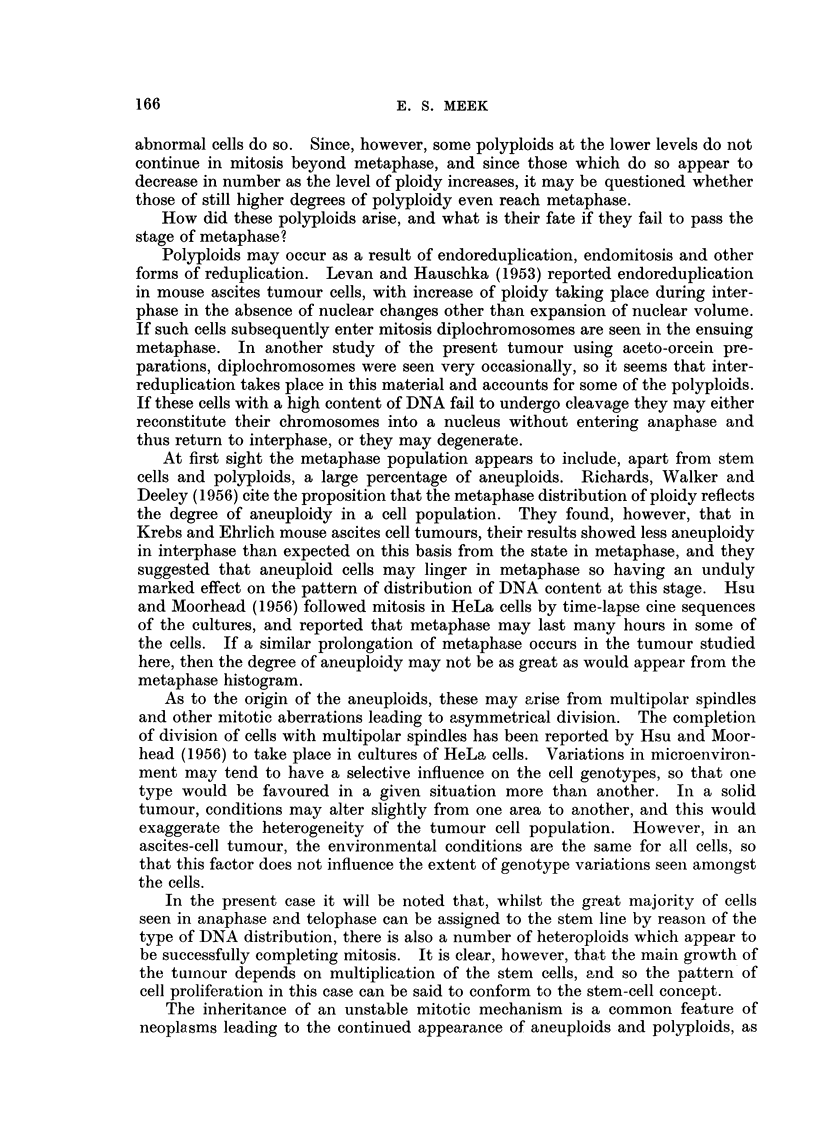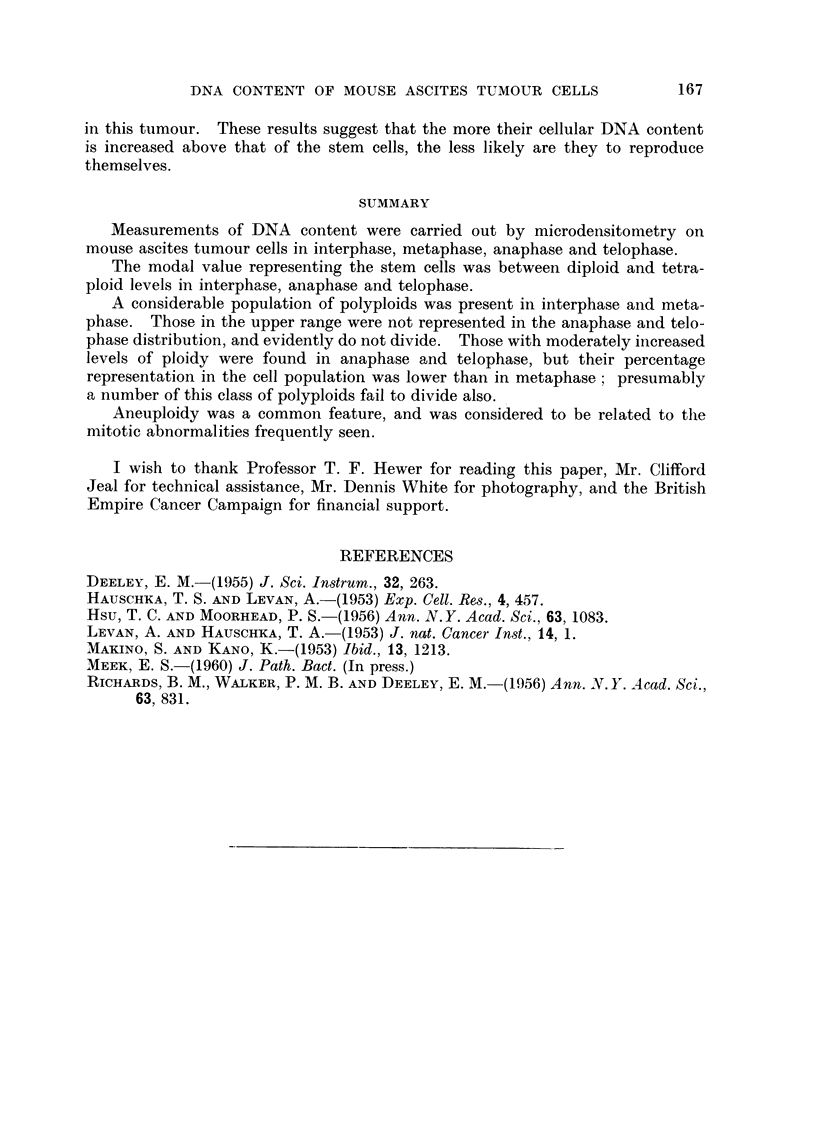# Deoxyribonucleic Acid Content of Mouse Ascites Tumour Cells in Interphase and Mitosis

**DOI:** 10.1038/bjc.1961.21

**Published:** 1961-03

**Authors:** E. S. Meek


### DEOXYRIBONUCLEIC ACID CONTENT OF MOUSE ASCITES TUMOUR CELLS IN INTERPHASE AND MITOSIS

E. S. MEEK

*From the Department of Pathology, University of Bristol*

Received for publication November 23, 1960

THE distribution of chromosome numbers and cellular deoxyribonucleic acid (DNA) values in both human and animal neoplasms show that those studied to date conform to the concept of the stem cell in tumour cell proliferation. The growth of a tumour depends on the progressive multiplication of its stem cells which form the majority of the cell population. Their genotype is characterised by their chromosome complement or DNA content ; this is not necessarily euploid.

However, in addition to the stem cells, there are often other neoplastic cells present with a degree of ploidy different from that of the stem line. Their frequency varies from tumour to tumour, and their chromosome number or level of DNA may exceed that of the stem cells, sometimes very considerably. Such cells do not appear to be associated with any particular stage of tumour development, and their continued presence in experimental neoplasms throughout numerous transplantations raises the question of their mode of origin. The possibilities are that they propagate by continued normal mitosis following their original appearance in the cell population, or that they arise *de novo* at intervals from the stem cells, and fail to reproduce.

The problem is not one of academic interest only, since the repeated clinical observation on human tumours of an initial satisfactory response to irradiation, chemotherapy or hormone treatment followed by subsequent resistance and renewed growth, points to the need for a more detailed knowledge of the genesis of the newly-resistant cell population.

At first sight the existence of tumour cells other than those of the stemline suggests a potential reservoir from which a new resistant strain might arise should it be favoured by the selective action of environmental alterations due to therapeutic measures. If so, the least likely candidates in the circumstances are the giant cells encountered from time to time, and one may doubt whether such cells are capable of giving rise to viable progeny at all. However, the status of those cells differing by only a relatively minor degree in ploidy level from that of the stem cells is worth further consideration although it may well be that the change involved is related to a point mutation only.

This paper describes the changes in distribution of cellular DNA values during interphase and at different stages of mitosis, in a mouse ascites-cell tumour, indicating that those cells with markedly increased DNA content do not reproduce themselves, but presumably arise as a result of mitotic aberrations, some of which are also responsible for aneuploidy.

### MATERIALS AND METHODS

Smears were made of a mouse ascites-cell tumour (T.2146), grown in an inbred laboratory strain of albino mice, taken at various times following transplantation. These were fixed immediately in methanol, stained with Feulgen, and microdensitometric measurements were then carried out on cells selected at random as described previously (Meek, 1960), using normal leucocytes as a standard of reference.

Estimations of DNA content, as judged by the absorption level, were made on cells in the intermitotic phase, and in metaphase, anaphase and telophase, taking 150 in each group, with anaphase and telophase counted together as one group.

### RESULTS

The distribution of cellular DNA content during interphase and in mitosis is seen in the histogram (Fig. 1). It is apparent that there is a considerable degree of both aneuploidy and polyploidy, and the wide range of values of nuclear DNA content is reflected in the microscopical appearances of the Feulgen-stained nuclei in smears.

These features are, however, common to tumours in general. The additional information gained in this study by microdensitometry is, first, that in spite of the wide scatter of cell types as shown by their DNA content, the cell population is dominated by the presence of a stem-cell line on which the growth of the tumour depends, and second that the polyploids in the higher ranges fail to divide.

Judged by the degrees of absorption in interphase, the major mode of DNA content lies between diploid and tetraploid levels. There is nevertheless a considerable proportion of the cells spread between tetraploid and octoploid values. Out of the 150 cells in interphase 39 (26 per cent) have DNA values higher than octoploid.

In metaphase, the major mode lies between tetraploid and octoploid levels, in a position corresponding to about twice the DNA content of the stem cells as shown by the interphase readings. Here again there is a very wide range of distribution of cellular DNA content with a quarter of the population beyond the octoploid level. Since the stem-cell line is appreciably below the tetraploid value this indicates the extent of polyploidy in the tumour. Of these, 17 have a DNA content greater than that of 12-ploidy.

The anaphase-telophase histogram shows a striking alteration in distribution. Ninety-three per cent of all these cells are situated in the diploid-to-tetraploid zone, and the major mode representing the stem cells is prominent ; this is in about the same position as in interphase. The remaining 7 per cent lie in the tetraploid-to-hexaploid region ; none exceed the hexaploid value.

### DISCUSSION

Variations in the amount of absorbing material present in single cells may be due to alterations in the DNA-Feulgen reaction, errors in measurement, synthesis of DNA during interphase, or to changes in the basic cell content of DNA with or without an alteration of the number of chromosomes.

FIG. 1.—Absorptiometric readings representing DNA content in 150 cells in interphase, 150 in metaphase, and 150 in anaphase and telophase combined. In the latter group, the readings are those taken from the two separated masses of DNA.

With regard to the first factor, it is not known whether variations take place in the stoichiometry of the Feulgen-DNA complex in these cells during mitosis ; this might be investigated by parallel measurements of the absorption of ultra-violet light on individual cells. As far as possible the errors inherent in micro-absorptiometry have been minimised by the use of the integrating microdensitometer designed by Deeley (1955).

In interphase, doubling of the amount of DNA occurs preparatory to mitosis and this leads to a spread in the pattern of cellular distribution, which is not related to genotypic differences. The wide distribution of readings in interphase is undoubtedly due in part to stem cells which had already completed their premitotic synthesis of DNA or were in the process of doing so. The results obtained in this study from the cells in interphase show a prominent mode surrounded by an extensive scatter of DNA values. The mode is taken to represent the stem cells responsible for the growth of the tumour, in accordance with the stem-cell concept of proliferation put forward by Hauschka and Levan (1953) and Makino and Kano (1953). In metaphase the mode is seen between tetraploid and octoploid values, in a position which agrees well with that expected from a doubling of the interphase stem-cell value. The metaphase content is then halved in normal mitosis when the cells proceed to the later stage, and here in anaphase and telophase the stem line is in about the same position as in interphase. Clearly the interphase group contains a large number of post-telophase cells which had not started synthesis of DNA. In view of the high mitotic rate it is interesting that there is a sufficient number of post-telophase cells to indicate the stem cell mode so clearly. These cells are responsible for the proliferation of the tumour.

In addition to the stem cells this tumour contains a number of aneuploids and polyploids. The presence of the former is suggested by the wide spread of values around the stem-line.

In both interphase and metaphase a quarter of the cell population is represented by DNA values exceeding the octoploid level, one cell in interphase having a DNA content equivalent to a state of 52-ploidy. The scatter between octoploid and 24-ploid levels is similar in these two stages, and one might expect that if the cells seen in metaphase completed mitosis, there would be a similar extension of readings in anaphase and telophase up to 12-ploidy. This is not so however, and the values in the latter group end at hexaploidy. The 17 metaphase cells with values greater than 12-ploidy are therefore not represented at all in the later stages of mitosis. However, those metaphase cells (22) with values in the region between octoploidy and 12-ploidy are reflected in the 10 cells in anaphase and telophase lying between tetraploid and hexaploid levels. The results suggest a failure on the part of the cells with high DNA content to continue in mitosis beyond metaphase. This failure appears to be relative to the level of ploidy, for those cells in the lower range of polyploidy evidently may enter anaphase or telophase, although apparently in fewer numbers, whereas those with still higher contents of DNA do not appear to proceed beyond metaphase at all.

It will be observed that there are three cells in interphase with a very high DNA content ; such giant cells are encountered occasionally in this tumour. None of corresponding size were seen amongst the metaphase cells. It is impossible to draw any firm conclusions from this observation alone as to whether these may enter mitosis or not, although one may doubt whether such grossly abnormal cells do so. Since, however, some polyploids at the lower levels do not continue in mitosis beyond metaphase, and since those which do so appear to decrease in number as the level of ploidy increases, it may be questioned whether those of still higher degrees of polyploidy even reach metaphase.

How did these polyploids arise, and what is their fate if they fail to pass the stage of metaphase?

Polyploids may occur as a result of endoreduplication, endomitosis and other forms of reduplication. Levan and Hauschka (1953) reported endoreduplication in mouse ascites tumour cells, with increase of ploidy taking place during interphase in the absence of nuclear changes other than expansion of nuclear volume. If such cells subsequently enter mitosis diplochromosomes are seen in the ensuing metaphase. In another study of the present tumour using aceto-orcein preparations, diplochromosomes were seen very occasionally, so it seems that inter-reduplication takes place in this material and accounts for some of the polyploids. If these cells with a high content of DNA fail to undergo cleavage they may either reconstitute their chromosomes into a nucleus without entering anaphase and thus return to interphase, or they may degenerate.

At first sight the metaphase population appears to include, apart from stem cells and polyploids, a large percentage of aneuploids. Richards, Walker and Deeley (1956) cite the proposition that the metaphase distribution of ploidy reflects the degree of aneuploidy in a cell population. They found, however, that in Krebs and Ehrlich mouse ascites cell tumours, their results showed less aneuploidy in interphase than expected on this basis from the state in metaphase, and they suggested that aneuploid cells may linger in metaphase so having an unduly marked effect on the pattern of distribution of DNA content at this stage. Hsu and Moorhead (1956) followed mitosis in HeLa cells by time-lapse cine sequences of the cultures, and reported that metaphase may last many hours in some of the cells. If a similar prolongation of metaphase occurs in the tumour studied here, then the degree of aneuploidy may not be as great as would appear from the metaphase histogram.

As to the origin of the aneuploids, these may arise from multipolar spindles and other mitotic aberrations leading to asymmetrical division. The completion of division of cells with multipolar spindles has been reported by Hsu and Moorhead (1956) to take place in cultures of HeLa cells. Variations in microenvironment may tend to have a selective influence on the cell genotypes, so that one type would be favoured in a given situation more than another. In a solid tumour, conditions may alter slightly from one area to another, and this would exaggerate the heterogeneity of the tumour cell population. However, in an ascites-cell tumour, the environmental conditions are the same for all cells, so that this factor does not influence the extent of genotype variations seen amongst the cells.

In the present case it will be noted that, whilst the great majority of cells seen in anaphase and telophase can be assigned to the stem line by reason of the type of DNA distribution, there is also a number of heteroploids which appear to be successfully completing mitosis. It is clear, however, that the main growth of the tumour depends on multiplication of the stem cells, and so the pattern of cell proliferation in this case can be said to conform to the stem-cell concept.

The inheritance of an unstable mitotic mechanism is a common feature of neoplasms leading to the continued appearance of aneuploids and polyploids, as in this tumour. These results suggest that the more their cellular DNA content is increased above that of the stem cells, the less likely are they to reproduce themselves.

SUMMARY

Measurements of DNA content were carried out by microdensitometry on mouse ascites tumour cells in interphase, metaphase, anaphase and telophase.

The modal value representing the stem cells was between diploid and tetraploid levels in interphase, anaphase and telophase.

A considerable population of polyploids was present in interphase and metaphase. Those in the upper range were not represented in the anaphase and telophase distribution, and evidently do not divide. Those with moderately increased levels of ploidy were found in anaphase and telophase, but their percentage representation in the cell population was lower than in metaphase ; presumably a number of this class of polyploids fail to divide also.

Aneuploidy was a common feature, and was considered to be related to the mitotic abnormalities frequently seen.

I wish to thank Professor T. F. Hewer for reading this paper, Mr. Clifford Jeal for technical assistance, Mr. Dennis White for photography, and the British Empire Cancer Campaign for financial support.

### REFERENCES

Deeley, E. M.—(1955) *J. Sci. Instrum.*, **32**, 263.
Hauschka, T. S. and Levan, A.—(1953) *Exp. Cell. Res.*, **4**, 457.
Hsu, T. C. and Moorhead, P. S.—(1956) *Ann. N.Y. Acad. Sci.*, **63**, 1083.
Levan, A. and Hauschka, T. A.—(1953) *J. nat. Cancer Inst.*, **14**, 1.
Makino, S. and Kano, K.—(1953) *Ibid.*, **13**, 1213.
Meek, E. S.—(1960) *J. Path. Bact.* (In press.)
Richards, B. M., Walker, P. M. B. and Deeley, E. M.—(1956) *Ann. N.Y. Acad. Sci.*, **63**, 831.